# The use of extended dose range film for dosimetric calibration of a scanning liquid‐filled ionization chamber electronic portal imaging device

**DOI:** 10.1120/jacmp.v8i1.2305

**Published:** 2007-02-28

**Authors:** Mohammad Mohammadi, Eva Bezak, Paul Reich

**Affiliations:** ^1^ Royal Adelaide Hospital Department of Medical Physics Adelaide Australia; ^2^ The University of Adelaide School of Chemistry and Physics Adelaide Australia; ^3^ Hamadan University of Medical Sciences Faculty of Medicine, Department of Medical Physics Hamadan Iran

**Keywords:** EDR2 film, electronic portal imaging, dose verification, transit dosimetry, treatment planning system

## Abstract

A scanning liquid‐filled ionization chamber electronic portal imaging device (SLIC‐EPID) and extended dose range (EDR2) films were used to evaluate transmitted dose profiles for homogeneous and inhomogeneous phantoms. Calibrated ionization chamber measurements were used to convert the pixel values acquired from the electronic portal images to dose. Because SLIC‐EPID was developed to have a uniform response for all liquid ionization chambers, the off‐axis dose values were reconstructed using a correction factor matrix, defined as the ratio of the relative EDR2 film and the corresponding EPID dose values measured in air. The transmitted dose distributions in the EPID detector layer were also modeled using a ${\text{Pinnacle}}^{3}$ treatment planning system (TPS: Philips Radiation Oncology Systems, Milpitas, CA). The gamma function algorithm was then used to assess agreement between transmitted dose distributions measured using a SLIC‐EPID and EDR2 film, and those calculated using the TPS. For homogenous and inhomogeneous phantoms, more than 90% agreement was achieved using gamma criteria of 2% and 3 mm and 3% and 2.5 mm respectively. Our results indicate that the calibration procedure proposed in the present study should be performed if SLIC‐EPID is to be used as a reliable two‐dimensional transmitted dosimeter for clinical purposes.

PACS numbers: 87.53.Tf, 87.53.Oq

## I. INTRODUCTION

Electronic portal imaging devices (EPIDs) are used to acquire digital images for patient positioning verification. Several studies have reported that camera‐based,^(^ ^1^ ^–^ ^4^ ^)^ scanning liquid‐filled ionization chambers (SLICs)^(^ ^5^ ^–^ ^13^ ^)^ and amorphous silicon (aSi)^(^ ^14^ ^–^ ^16^ ^)^ EPIDs can be used for dosimetry. The dosimetric procedures with EPIDs can generally be divided into three parts:

- Dosimetric calibration
- Pretreatment assessment
- *In vivo* dosimetric verifications

The dosimetric calibration procedure includes the conversion of EPID raw pixel values into dose. The term “pretreatment dose verification” is defined as the verification of dose delivery in a phantom before the first radiotherapy session.^(4)^ *In vivo* dose verification is the actual patient dose measurement using EPIDs.

Two methods using measured portal dose distributions called “transmitted dose maps” have been developed to verify the *in vivo* dose delivered to the patient. In the first approach, the transmitted dose maps^(^ ^10^ ^,^ ^17^ ^–^ ^21^ ^)^ are back‐projected to obtain either exit dose maps or midplane dose maps.^(^ ^3^ ^,^ ^10^ ^,^ ^17^ ^–^ ^19^ ^,^ ^22^ ^)^ In the second approach, the transmitted dose maps measured using EPIDs are compared with predicted portal dose images, calculated using a treatment planning system (TPS) or another method leading to portal doses.^(^ ^8^ ^,^ ^23^ ^–^ ^28^ ^)^

Although several empirical^(^ ^11^ ^,^ ^29^ ^)^ and mathematical^(^ ^8^ ^,^ ^16^ ^,^ ^30^ ^)^ approaches for EPID dosimetry have been developed, the accuracy of empirical methods for radiation primary fluence maps is within 3%–4%^(^ ^11^ ^,^ ^29^ ^)^ and decreases for either pretreatment or *in vivo* dosimetry. As compared with primary fluence map verifications, mathematical techniques for dose delivery verification require specific algorithms for patient or sophisticated phantoms.^(^ ^16^ ^,^ ^30^ ^,^ ^31^ ^)^ The application of EPID for routine dosimetry in clinics is thus infrequent.

The aim of the present work was to verify an empirical dosimetric calibration method developed for SLIC‐EPIDs in the presence of homogeneous and inhomogeneous phantoms. Electronic portal images (EPIs) acquired using a SLIC‐EPID were converted to dose using calibrated ionization chamber measurements for a range of dose rates on the central axis of the radiation beam. To calibrate the EPID in the off‐axis areas, a correction factor matrix (CFM) was defined as the ratio of extended dose range (EDR2) film and EPID dose values in no‐phantom (air) conditions. To verify this calibration method, the gamma function algorithm was used to compare the transmitted dose maps measured by SLIC‐EPID with those measured using EDR2 films and those calculated by the ${\text{Pinnacle}}^{3}$ TPS for a range of homogeneous and inhomogeneous phantoms.^(32)^

## II. METHODS

### A. Materials

#### A.1 Linear accelerator

A Varian 600CD linear accelerator treatment system (LINAC) equipped with a SLIC‐EPID (LC250 PortalVision MK2: Varian Oncology Systems, Palo Alto, CA) was used to perform all measurements. This LINAC produces a 6‐MV photon beam with dose rates from 100 to 600 monitor units (MUs) per minute. All data acquisition was performed using a repetition rate of $300\;\text{MU}\min^{-1}$, with 1 MU corresponding to a calibrated dose delivery of 1 cGy under reference conditions.

#### A.2 SLIC‐EPID

The SLIC‐EPID consisted of 256×256 ion chambers filled with an organic fluid (2,2,4‐trimethylpentane: Merck, Darmstadt, Germany) with reasonable electron mobility.^(33)^ The volume of each ionization chamber was $1.27\times 1.27\times 1\;{\text{mm}}^{3}$. Stainless steel 1 mm thick acted as a build‐up material. A polarizing voltage of 400 V was applied to each row. The ionization chamber currents in all columns were measured and recorded as pixel values. To increase the quality of EPIs for dosimetric tasks, all EPIs were acquired in fast readout and full resolution mode.^(13)^ To reduce the statistical fluctuation of EPID response, each EPI used in this study was obtained as the average of two independent and consecutively acquired images with a pixel‐value standard deviation of less than 1%.^(5)^ Additional physical characteristics of a SLIC‐EPID have been explained extensively in the literature.^(^ ^5^ ^,^ ^33^ ^,^ ^34^ ^)^

#### A.3 EDR2 films

The EDR2 film (Kodak Extended Dose Range 2: Eastman Kodak, Rochester, NY) was used as a reference two‐dimensional (2D) dosimeter. The small size and very fine monodispersedgrain cubic microcrystal used in the EDR2 structure makes it a very slow‐speed film as compared with other conventional films; it is nearly energy independent for dose values of less than 5 Gy.^(^ ^35^ ^–^ ^41^ ^)^ For rapid‐processing purposes, the EDR2 film is covered with double emulsion layers on a 0.18‐mm ester base.^(42)^

Dose values obtained from EDR2 film measurements have been reported to agree with ionization chamber measurements to within 1%–2%.^(^ ^41^ ^,^ ^43^ ^–^ ^45^ ^)^ In the present study, EDR2 film was used for SLIC‐EPID 2D calibration for dosimetric purposes. In addition, several EDR2 films were used to measure relative transmitted doses for a range of homogeneous and inhomogeneous phantoms.

#### A.4 Treatment planning system

A commercial TPS, ${\text{Pinnacle}}^{3}$ (v6.2b: Philips Radiation Oncology Systems, Milpitas, CA) was used to predict transmitted dose delivered to the EPID sensitive layer. The ${\text{Pinnacle}}^{3}$ TPS calculates the dose using a collapsed cone convolution superposition algorithm.^(^ ^46^ ^,^ ^47^ ^)^

#### A.5 Phantoms

A range of $30\times 30\times 1‐{\text{cm}}^{3}$ white water slabs, RW3 ($\rho =1045\;g/{\text{cm}}^{3}$; PTW Freiburg, Freiburg, Germany), were used as homogeneous phantoms. Homogeneous phantoms 6‐, 10‐, 16‐, and 20‐cm in thickness were used.

**Figure 1:**
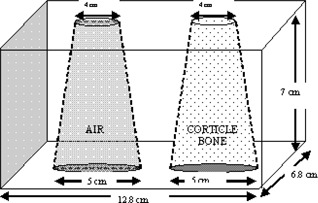
An inhomogeneous phantom consisting of bone and air

An inhomogeneous phantom was created by inserting two semi‐conical objects of different density into a cylindrical phantom made of solid water (Fig. 1). The original diameter of the cylindrical phantom was 30 cm, and the height was 7 cm. In the present work, a $6.8\times 12.8‐{\text{cm}}^{2}$ area of the cylindrical phantom, including two semi‐conical holes as shown in Fig. 1, was used as an inhomogeneous phantom. The left insertion was made of lung‐equivalent material $\left(\rho =1.20\;g/{\text{cm}}^{3}\right)$, and the right insertion was made of cortical bone–equivalent material $\left(\rho =1.75\;g/{\text{cm}}^{3}\right)$.

#### A.6 Image processing tool

All image processing and dose comparison procedures were performed using in‐house codes written in MATLAB 6.5 (MathWorks, Natick, MA).

### B. Methods

#### B.1 EPID dose maps

The procedure for dosimetric calibration of the SLIC‐EPID for radiation primary fluence verification has been discussed in depth elsewhere.^(^ ^45^ ^)^ Briefly, several EPIs were acquired for a range of source‐to‐EPID distances (SEDs). No phantom was used for these EPIs. To reach electronic equilibrium at the EPID detector layer, 5 mm of RW3 white water was placed as an extra build‐up layer on the surface of the EPID cover for all EPID measurements.^(^ ^48^ ^)^ An 8×8‐pixel matrix in the central part of each EPI was selected and averaged to give the pixel value on the central axis. The area represented by this pixel array was $0.72\times 0.72\;{\text{cm}}^{2}$ at the isocenter and $1\times 1\;{\text{cm}}^{2}$ at the EPID detector layer. This array size was chosen to provide sufficient spatial resolution while minimizing statistical fluctuations in pixel response. The relationship between the EPI pixel values and the dose measured using a calibrated ionization chamber positioned in a slab of solid water was investigated on the central axis of the radiation beam. The equation

(1) $$D=a{\left(PV\right)}^{b},$$

where *D* is the dose delivered to the point of interest on the central axis and *PV* is the EPI pixel values, was used. The parameters *a* and *b* are derived from a line of best fit and depend on the setting of the EPI acquisition, the LINAC repetition rate, and the EPID calibration procedure.

To calibrate the SLIC‐EPID as a 2D dosimeter, EPIs were acquired at a source‐to‐surface distance of 140 cm for a $17\times 17‐{\text{cm}}^{2}$ field size. An EDR2 film was also irradiated with 588 MUs under the same conditions. The irradiated film was processed using an automatic Agfa Curix 160 processor (Agfa, Mortsel, Belgium) and scanned using a Vidar scanner (Vidar Systems Corporation, Herndon, VA). Non‐uniform response of the scanner, especially in the horizontal axis, was found and corrected using a blank (nonirradiated) EDR2 film. To verify the EDR2 film response, its inplane and crossplane profiles were also compared with corresponding ionization chamber profiles obtained with a water‐tank dosimetry system (Wellhöfer Dosimetrie: Scanditronix Medical, Schwarzenbruck, Germany) under the same conditions. A CFM was then defined to relate 2D EPI relative dose values to corresponding EDR2 film relative dose values:

(2) $${\text{CFM}}_{i,j}=\frac{D_{i,j}(EDR2\;\;film)}{D_{i,j}(EPI)},$$

where $D_{i,j}$ (EDR2 film) and $D_{i,j}$ (EPID) are pixel values obtained from the EDR2 film and from the EPID measurements respectively. With the use of the CFM, the relative absorbed dose in water at all points of acquired EPID images was calculated as follows:

(3) $$D_{i,j}\;(EPID\;Corrected)=D_{i,j}\;(EPID\;Measured)\times {\text{CFM}}_{i,j}.$$

A radiation beam fluence calibrated using this method has been shown to be within 1% agreement with EDR2 film measurements for a range of open‐field and wedged‐field conditions.^(^ ^45^ ^)^

#### B.2 Evaluation of EPID dose values for homogeneous phantoms

A series of EPIs were acquired for homogeneous phantoms consisting of RW3 layers of 6‐, 10‐, 16‐, and 20‐cm thickness, using isocentric technique for a $10\times 10\;{\text{cm}}^{2}$ nominal field size at a SED of 140 cm. Acquired EPIs were converted to the transmitted dose maps using the above‐mentioned calibration procedure. The EDR2 films were irradiated with 3 Gy and 5 Gy (588 MUs and 980 MUs) for 6‐ and 10‐cm and for 16‐ and 20‐cm phantom thicknesses respectively, under the same conditions as described for the EPIs. The transmitted dose maps measured using EDR2 film were compared with those measured using the SLIC‐EPID (after CFM application). Because of the different pixel size of the scanned EDR2 films, bilinear interpolation was used to rescale the EDR2 film images to EPI pixel size. The EPID and EDR2 film dose profiles were both normalized about a 7×7 matrix at the calibration point. The gamma function algorithm developed by Low et al.^(32)^ was then used to assess agreement between the EDR2 film and the corrected EPID portal dose maps, using distance to agreement (DTA) and dose difference $\left(\Delta D_{\max}\right)$ criteria of 2.54 mm (2 pixels) and 1% respectively. In addition, a 2.5‐mm margin at the edge of the EPID and film images was excluded from the gamma analysis because of translational and rotational misalignment errors.

#### B.3 Evaluation of dose maps measured using a SLIC‐EPID for inhomogeneous phantoms

**Figure 2:**
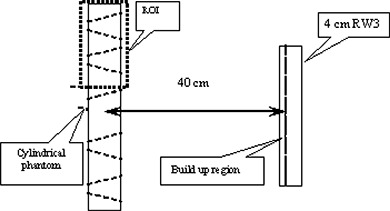
A sagittal view of a scanned inhomogeneous phantom used in the treatment planning system, where ROI is the region of interest

The transmitted dose maps were measured using SLIC‐EPID for an inhomogeneous phantom (see Fig. 1). The thickness of the phantom was increased to 30 cm from 7 cm using additional RW3 layers placed on the top and bottom of the phantom. Under the same conditions, EDR2 films were also irradiated with 588 MUs and 980 MUs for 7‐cm and 17‐cm and for 20‐cm and 30‐cm phantom thicknesses respectively. To predict the transmitted dose distribution, the cylindrical phantom, a 4‐cm‐thick RW3 slab representing the modeled EPID, and a 40‐cm air gap between centre of the phantom and the slab were scanned using a computed tomography simulator (Philips Medical Systems). Fig. 2 shows a schematic sagittal view of the extended phantom. More information about predicted portal dose images for the extended phantom is published elsewhere.^(49)^ The slice thickness used in this study was 5 mm in the superior–inferior direction. In the TPS, the thickness of the phantom was varied to 17, 20, and 30 cm by converting the air density to the water‐equivalent material added to the CT data for the entrance and exit surface of the inhomogeneous cylindrical phantom. The transmitted dose was calculated at $D_{\max}$ in the modeled EPID.

The EPI, EDR2, and TPS dose maps were aligned at the 50% isodose lines. The gamma function algorithm was used to compare the dose values from EPID, EDR2 film, and TPS. A DTA of 2.54 mm (2 pixels) was used, and the $\Delta D_{\max}$ was varied from 1% to 2% for the EDR2 film–EPID comparison. Because of the difference between the measurement and calculation procedures, a maximum dose difference criterion of 3% was selected for the TPS–EPID comparison.

## III. RESULTS

### A. Fluence map

**Figure 3:**
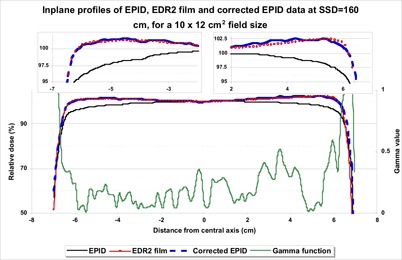
Typical beam profiles measured using extended dose range (EDR2) film, an electronic portal imaging device (EPID), and corrected EPID at 140 cm source‐to‐surface distance for a $10\times 12\;{\text{cm}}^{2}$ field size. The corresponding gamma values are shown for 2.54 mm and 1% criteria

Fig. 3 shows typical line profiles of a radiation primary fluence measured using EDR2 film and SLIC‐EPID (before and after applying CFM) and the result of the corresponding gamma function with DTA and $\Delta D_{\max}$ criteria (2.5 mm and 1% respectively). The X axis represents the distance from the central axis, and the Y axis represents relative dose values. The gamma values are also shown as a secondary Y axis. A significant difference was observed between line profiles measured using EPID and EDR2 film in off‐axis areas. After applying the CFM, we found excellent agreement (more than 96%) between the beam fluence maps measured using EPID and those measured using EDR2 film.

### B. Evaluation of transmitted dose for homogenous phantom

**Figure 4:**
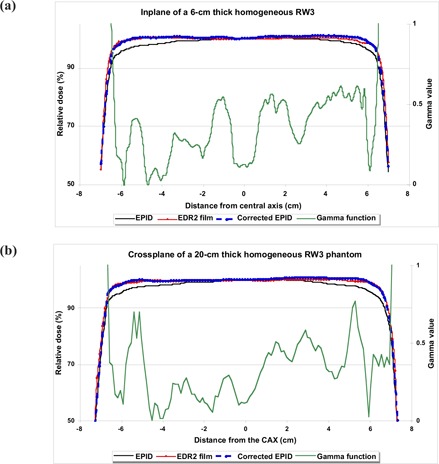
The inplane and crossplane relative dose profiles measured using an electronic portal imaging device (EPID), extended dose range (EDR2) film, and corrected EPID (using a correction factor matrix), with corresponding gamma profiles using 1% and 2.54 mm criteria for homogeneous phantom thicknesses of (a) 6 cm and (b) 20 cm

**Figure 5:**
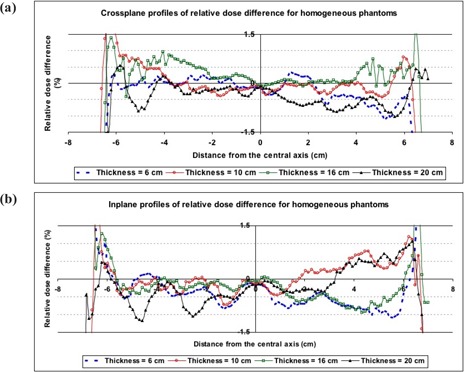
The (a) crossplane and (b) inplane of relative dose difference between corrected electronic portal images and extended dose range (EDR2) film for 6‐, 10‐, 16‐, and 20‐cm homogeneous phantom thicknesses

Fig. 4 shows typical inplane and crossplane relative dose profiles for the EPID, EDR2 film, corrected EPID, and gamma profiles (DTA=2.5 mm, ΔD=1%) for phantom thicknesses of 6 and 20 cm. As with the fluence maps, a significant difference (maximum of 4.6%) was observed between EDR2 film and EPI dose values in off‐axis areas before CFM correction. After correcting the EPID transmitted dose measurements with the CFM, the gamma function results showed good agreement (more than 90%) between the EDR2 film dose and the EPID dose values for 1% and 2.5 mm criteria. The corresponding relative dose difference between corrected EPID and EDR2 film were found to be less than 1% in the central part of the radiation field (see Fig. 5). However, in the edge of the field, several differences of more than 1% were observed. Excluding the penumbra region, no systematic variation in dose difference profiles was observed.

**Figure 6:**
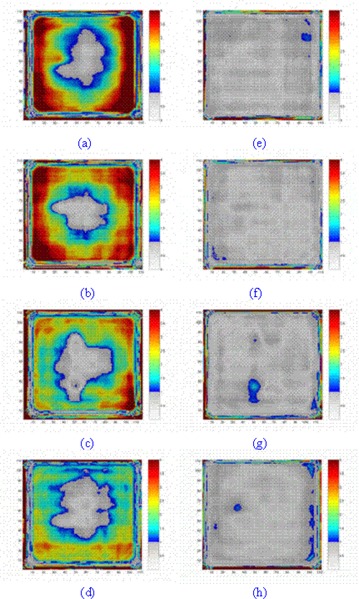
The agreement between extended dose range (EDR2) film and electronic portal imaging device (EPID) dose values (a–d) before correction and (e–f) after correction for 6‐, 10‐, 16‐, and 20‐cm phantom thicknesses at a source‐to‐EPID distance of 140 cm for a $10\times 10\;{\text{cm}}^{2}$ field size. The gamma maps were calculated for 2.5 mm and 1% criteria

Fig. 6 shows the agreement between relative transmitted doses for EDR2 film and corrected EPI before and after application of the CFM. Before the CFM was used, only the central regions of the gamma maps were in agreement. Application of the CFM significantly increased the area of agreement, as indicated by the gamma maps. Table 1 shows additional information about the agreement percentage between EDR2 film and corrected EPI.

**Table 1:** The percentage agreement between dose values obtained from extended dose range (EDR2) film and a scanning liquid‐filled ionization chamber electronic portal imaging device (SLIC‐EPID), using the gamma function for homogeneous phantoms

Phantom thickness (cm)	Before correction	Agreement (%) (DTA=2.5 mm, ΔD=1%) After correction	Excluding 2.5‐mm edge
6	25.22	89.12	95.90
10	16.66	90.00	95.99
16	16.10	87.08	93.63
20	21.78	83.51	90.89

The use of the CFM increases the percentage agreement between the dose distributions measured using EDR2 film and EPID from 20%–25% to approaching 90%–95% in each case. Although several nonsystematic differences were observed in the gamma maps, the disagreement occurred mainly within 2.5 mm of the edge of those maps and is most likely attributable to misalignment between the dose maps. The agreement between the two measured data sets decreases with the increase in phantom thickness.

### C. Evaluation of transmitted dose for inhomogeneous cylindrical phantoms

**Figure 7:**
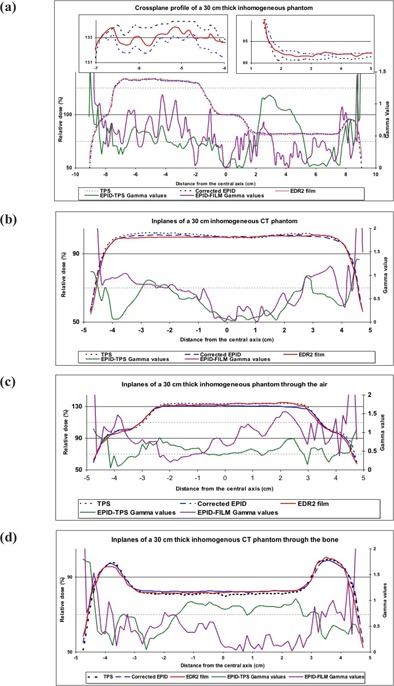
(a) Crossplane, (b) inplane, and off‐axis inplane profiles passing through (c) air and (d) bone inhomogeneities for the extended dose range (EDR2) film and the treatment planning system, and the corrected electronic portal image doses and the corresponding gamma values for 3% and 2.5 mm criteria for a 30‐cm‐thick phantom

Fig. 7 shows typical crossplane, inplane, and off‐axis inplanes passing through the inhomogeneities and corresponding gamma values for EDR2 film–EPID and TPS–EPID comparisons for a 30‐cm‐thick inhomogeneous phantom. The Fig. 7 results show that the CFM, defined for in‐air conditions, can be used for complex conditions with reasonable accuracy. Gamma function assessment also shows that corrected EPID dose values agree in the main with those measured using EDR2 film. However, several nonsystematic discrepancies were observed in the region‐of‐interest edges, the inhomogeneity boundaries, and the homogeneous regions. For all inplane profiles, an increase in disagreement was observed towards edges. No systematic variation in EDR2 film–EPID gamma values was observed in crossplanes. When comparing TPS line profiles with corrected EPID and with EDR2 film, the most discrepancy was found in the TPS inplane profiles. This discrepancy was not observed for other off‐axis profiles passing through the inhomogeneous regions.

Because of the misalignment errors in the dose profiles, significant differences were observed in high dose gradient regions, near the edge corresponding to the inhomogeneities. The relative dose differences for EDR2 film–EPID and TPS–EPID dose maps were found to be within 2% and 3% respectively. When comparing the in‐axis and off‐axis inplanes of relative dose difference between TPS and EPID, the maximum and minimum relative dose difference was observed for air and bone inhomogeneities respectively.

**Figure 8:**
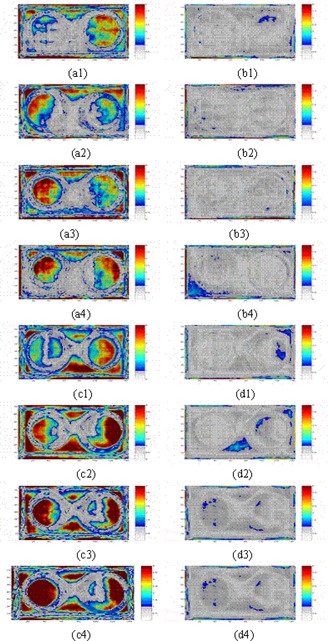
Gamma maps comparing extended dose range (EDR2) film and an electronic portal imaging device (EPID) (a1–a4) before correction, and (b1–b4) after correction with 2% and 2.5 mm criteria, and gamma maps comparing the treatment planning system and an EPID (c1–c4) before correction, and (d1–d4) after correction with 3% and 2.5 mm criteria, for phantom thicknesses of 7, 17, 20, and 30 cm respectively

Fig. 8 shows typical gamma maps for EDR2 film and TPS relative dose distributions. As panels a1–a4 and c1–c4 show, the central part of radiation field and the edge of inhomogeneities are in disagreement before application of the CFM. After correction of the EPID dose maps, the radiation fields are almost entirely in agreement, as shown in panels b1–b4 and d1–d4.

Although several disagreements between EPID and EDR2 film dose distributions were seen in the homogeneous and air inhomogeneity regions, no significant discrepancies were observed in the bone inhomogeneity region. For instance, gamma values larger than 1 were observed in the homogeneous areas for 7‐ and 17‐cm thicknesses. In addition, in the air inhomogeneity region, differences were observed for the 17‐ and 30‐cm thicknesses. For the bone inhomogeneity, the only spot where the gamma function was larger than 1 was observed for the 7‐cm phantom thickness.

For the TPS–EPID comparison, the relative dose difference between EPID and TPS data sets were larger than those for the EDR2 film–EPID comparison. The gamma values increased with the increase in phantom thickness. In the air inhomogeneity region, an increase in the gamma values, indicating more disagreement, was observed with increase in phantom thickness. No significant increase in gamma values was observed for bone inhomogeneity with increase in phantom thickness. Disregarding the edges of the radiation field, no significant difference was observed in the homogenous area and in the bone inhomogeneity after application of the CFM. The average gamma value and related standard deviation for the homogenous area and for air and bone inhomogeneities were 0.58±0.14, 0.46±0.12, and 0.59±0.18 respectively.

Tables 2 and 3 show the percentage agreements for the film–EPID and TPS–EPID comparisons, before and after application of the CFM and discarding 2.5‐mm edges for a dose difference criterion of 2% and 3% respectively. For the film–EPID comparison, no systematic variation of gamma scores was found with increase in phantom thickness. However, after applying the CFM, a decrease in gamma scores was generally observed with increase in phantom thickness. A significant increase (44.9%, one standard deviation) was observed in agreement between reference and evaluated transmitted dose maps for the film‐EPID comparison. Ignoring 2.5‐mm edges in the radiation field increased the agreement by ~7%. Good agreement (more than 94%) was observed using 3% and 2.5 mm gamma criteria in all cases after ignoring 2 pixels around the edges. In these cases, application of the CFM increased the agreement by 60%.

**Table 2:** The percentage agreement between dose maps obtained from extended dose range (EDR2) film and an electronic portal image (EPI), using the gamma function for inhomogeneous phantoms, where CFM is the correction factor matrix

Phantom thickness (cm)	Before CFM	Agreement (%) (DTA=2.5 mm, ΔD=2%) After CFM	Discarding 2.5‐mm edges
7	46.25	91.66	97.25
17	47.47	89.41	96.36
20	35.28	89.50	96.06
30	46.71	84.70	92.19

**Table 3:** The percentage agreement between dose maps obtained from the treatment planning system (TPS) and an electronic portal image (EPI), using the gamma function for inhomogeneous phantoms, where CFM is the correction factor matrix

Phantom thickness (cm)	Before CFM	Agreement (%) (DTA=2.5 mm, ΔD=3%) After CFM	Discarding 2.5‐mm edges
7	32.68	89.76	96.13
17	31.82	89.49	96.39
20	24.17	89.01	95.94
30	27.41	87.46	94.08

**Figure 9:**
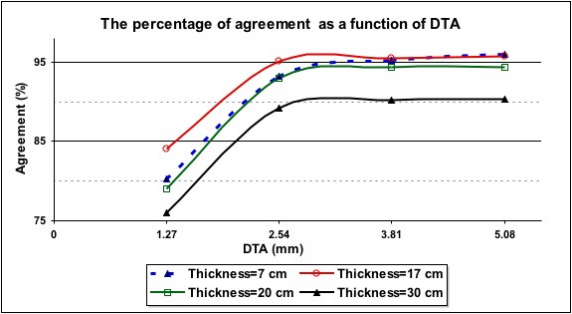
The percentage agreement between relative dose values calculated using the treatment planning system (TPS) and those measured using an electronic portal imaging device [EPID—after application of a correction factor matrix (CFM)] versus distance to agreement (DTA) for an inhomogeneous phantom of various thicknesses. The $\Delta D_{\max}$ was 2.5% in all cases

To find the maximum misalignment between dose maps obtained from the SLIC‐EPID and the TPS, the percentage agreement was investigated for a range of gamma function criteria. The DTA was varied from 1 to 4 pixels (1.27 mm to 5.08 mm) and the $\Delta D_{\max}(2.5\%)$ was kept constant for all inhomogeneous phantoms. Fig. 9 shows the results. A significant increase in agreement was observed with an increase in DTA from 1 pixel (1.27 mm) to 2 pixels (2.45 mm); agreement remained constant with the increase of DTA from 2 pixels to 3 or 4 pixels.

## IV. DISCUSSION

### A. Calibration method

Although several methods have been introduced for EPID dosimetric calibration, in all empirical calibrations,^(^ ^9^ ^,^ ^11^ ^,^ ^29^ ^)^ ionization chamber measurements have been used to correct transmitted or fluence dose maps in off‐axis areas. The ionization chamber measurements cannot be used to create comprehensive 2D correction factors. On the other hand, they can be useful for line profile verification. In addition, because of the size of the ionization chamber, the pixel size of ionization chamber measurements is larger than 2 mm. The size of EPID pixel values ranges from $0.25\;{\text{mm}}^{2}$ to $1.27\;{\text{mm}}^{2}$, and so re‐scaling of the data is required, decreasing the accuracy of measurement. In contrast, the pixel size of film measurement in the optimum scanning condition (300 dpi) is close to 0.3 mm and can be improved to 0.05 mm. Therefore, despite the accuracy of ionization chamber measurements, they are not the most suitable for a 2D comprehensive calibration. As a result, from the available 2D dosimeters, EDR2 film was found to be the best option for the present study. Even though EDR2 film is not a completely energy‐independent tool, its accuracy for this study was sufficient.

The results of the present work show that the CFM, measured in no‐phantom condition, can be used to correct EPI dose values measured in the presence of homogeneous and inhomogeneous phantoms. However, for the inhomogeneous phantoms a 0.5%–1% increase in the $\Delta D_{\max}$ criterion (for a fixed DTA) is required to reach results equivalent to those in homogeneous phantoms. The differences between EDR2 film and EPID dose values arise from the misalignment between EDR2 films and EPID images that are invariably present before and after the CFM is applied. In several cases, film positioning errors during irradiation and scanning led to a rotation of less than 1 degree in EDR2 film. This rotation increased the disagreement between measurements in the edge of the radiation fields. In addition, EDR2 films are not completely energy‐dependant.^(50)^

In a report of Parsaei et al.,^(11)^ a flattening material (10‐cm homogeneous phantom) was used to remove the horns in the beam profiles at the position of the EPID. Those authors reported an agreement of 3% between ionization chamber and EPID dose values. However, in our work, an empirically derived off‐axis correction factor (CFM) was used to restore the horns in the EPID beam profiles within an accuracy of 1% relative to EDR2 film dose measurements.

### B. Evaluation of transmitted dose for homogenous and inhomogeneous phantoms

According to the measured transmitted doses for homogeneous and inhomogeneous phantoms (Tables 1–3), the main reasons for the decrease in agreement between reference and evaluated dose maps are the increase in scattered radiation because of the increase in phantom thickness and the decrease in primary radiation measured in the EPID detector layer for thicker phantoms.

The gamma values observed for inhomogeneous regions were different from those for the same irradiation conditions in the homogenous cases. The different behavior in the inhomogeneous regions can be explained by the physical characteristics of inhomogeneities. In this case, the physical characteristics of bone are closer to those of soft tissue than to those of air, because the beam quality passing through bone is higher than that passing through air. Moreover, the calibration procedure proposed here is performed for a radiation fluence map. Because of beam hardening, the off‐axis radiation spectrum is changed in passing through phantoms. In passing through the various materials, the photon quality therefore produces different responses—especially in the EDR2 films.

We observed a significant difference in the radiation field edges. Although the gamma function tool can to some extent control misalignment, the discrepancy in the edge area is large and cannot be controlled with conventional DTA values. We chose to exclude the disagreement in the peripheral region of interest by excluding 2 pixels (2.54 mm) from the gamma function analysis. However, because of focusing on the central part of radiation field, pixel exclusion should not affect the results significantly.

Gamma function assessment is a suitable tool for comparing 2D dose maps obtained from either measurements or calculation. As Fig. 9 shows, identifying the magnitude of misalignment is also possible. In all of our cases, the maximum misalignment was observed to be 2 pixels, because no significant variation of agreement was observed with an increase of DTA of more than 2 pixels. However, the gamma function is not able to discern between positive and negative dose differences, nor to discern the direction of translational misalignments.

## V. CONCLUSION

Although SLIC‐EPIDs are calibrated during routine manufacturer‐designed calibration to generate uniform response in a flood radiation field, they can also be used for 2D dosimetry provided that an appropriate dosimetric calibration is used. The CFM developed in the present work is essential for a 2D dosimetric calibration of the EPID. Application of the CFM accurately reconstructs the horns of radiation profiles removed during calibration for portal imaging mode. The consistency between transmitted dose distributions measured using a SLIC‐EPID (corrected using a corresponding CFM) or EDR2 film and those calculated using a TPS showed that the SLIC‐EPID can be used as a reliable 2D dosimeter for pretreatment assessments. The mentioned calibration method is also independent of EPID type and EPI acquisition settings. Because of the limitation in EPI acquisition time, further investigation is required to evaluate the response of SLIC‐EPID for absolute dosimetry.

The SLIC‐EPIs acquired in fast readout and full resolution mode can be used as a reliable relative pretreatment 2D portal dosimeter for clinical purposes. This method can be used for traditional radiation therapy, three‐dimensional conformal radiation therapy, and step‐and‐shoot intensity‐modulated radiation therapy. Because the EPI acquisition time and pixels depend on the LINAC repetition mode,^(45)^ further investigation is required to use a SLIC‐EPID as a transmitted dosimeter for dynamic intensity‐modulated radiation therapy.

## ACKNOWLEDGMENT

The authors express their gratitude to the referees for fruitful comments and comprehensive editing. In addition, author MM gratefully acknowledges the award of a scholarship for PhD study from the Iranian Ministry of Health and Medical Education.
